# Meropenem-Vaborbactam Activity against U.S. Multidrug-Resistant *Enterobacterales* Strains, Including Carbapenem-Resistant Isolates

**DOI:** 10.1128/spectrum.04507-22

**Published:** 2023-01-09

**Authors:** Dee Shortridge, Valerie Kantro, Mariana Castanheira

**Affiliations:** a JMI Laboratories, North Liberty, Iowa, USA; Universidad de Buenos Aires, Facultad de Farmacia y Bioquímica

**Keywords:** CRE, U.S. surveillance, meropenem-vaborbactam, surveillance studies

## Abstract

Carbapenems are a common first-line therapy for serious Gram-negative infections, but carbapenem-resistant *Enterobacterales* (CRE) isolates have become an urgent health concern. Klebsiella pneumoniae serine carbapenemases (KPCs) now have been disseminated worldwide and are endemic in many hospitals globally. Isolates producing metallo-β-lactamases (MBLs) or class D OXA-48 carbapenemases are also increasingly common in Europe, although they are less common in the United States. Meropenem-vaborbactam is a combination of the carbapenem meropenem and vaborbactam, which is a β-lactamase inhibitor with activity against serine carbapenemases, including KPC-producing isolates. We examined the susceptibility of U.S. multidrug-resistant (MDR) isolates to meropenem-vaborbactam. A total of 1,697 MDR *Enterobacterales* isolates were collected in 31 U.S. medical centers in 2016 to 2020. Susceptibility testing was performed using the Clinical and Laboratory Standards Institute (CLSI) broth microdilution method. Whole-genome sequencing was performed for all CRE strains (MIC values of >2 mg/L for imipenem or meropenem). The rate of susceptibility of all MDR *Enterobacterales* strains to meropenem-vaborbactam was 99.1%, and 86.2% of the isolates were susceptible to meropenem. There were 222 CRE isolates (13.1%). KPC was the most common carbapenemase (81.1%). Thirteen CRE isolates produced NDM (*n *= 7), VIM (*n *= 3), and/or OXA-48-like (*n *= 4) carbapenemases; 29 CRE isolates (13.1%) had no detected carbapenemase. The rate of susceptibility of all CRE strains to meropenem-vaborbactam was 93.2%, and the rate of susceptibility of the KPC-producing isolates to meropenem-vaborbactam was 98.9%. The primary carbapenemase in the United States continues to be KPC, while MBL and OXA-48-like carbapenemases remain uncommon. Overall, the rate of susceptibility of these U.S. MDR organisms to meropenem-vaborbactam was 99.1%, indicating that meropenem-vaborbactam is a valuable treatment option for Gram-negative infections caused by U.S. MDR organisms.

**IMPORTANCE** Carbapenems are a common first-line therapy for serious Gram-negative infections, but CRE isolates have become an urgent health concern. Meropenem-vaborbactam is a combination of the carbapenem meropenem and vaborbactam, which is a β-lactamase inhibitor with activity against serine carbapenemases, including KPC-producing isolates. We examined the susceptibility of U.S. MDR Gram-negative isolates to meropenem-vaborbactam. A total of 1,697 U.S. MDR *Enterobacterales* isolates collected in 2016 to 2020 were tested. Susceptibility testing was performed using the CLSI broth microdilution method. Whole-genome sequencing was performed for all CRE strains (MIC values of >2 mg/L for imipenem or meropenem). The rate of susceptibility of all MDR *Enterobacterales* strains to meropenem-vaborbactam was 99.1%, and 86.2% of the isolates were susceptible to meropenem. A total of 13.1% of the isolates were CRE strains, and KPC was the most common carbapenemase. Overall, the rate of susceptibility of these U.S. MDR organisms to meropenem-vaborbactam indicates that meropenem-vaborbactam is a valuable treatment option for Gram-negative infections caused by U.S. MDR Gram-negative pathogens.

## INTRODUCTION

Infections caused by antimicrobial-resistant bacterial pathogens were globally associated with 4.95 million deaths in 2019, and resistance to first-line therapies is continuing to increase (1). Carbapenems have been a common first-line therapy for serious Gram-negative infections; as a result, carbapenem-resistant *Enterobacterales* (CRE) isolates are a growing global concern (2, 3). Among the carbapenemases detected in *Enterobacterales* species, Klebsiella pneumoniae serine carbapenemases (KPCs) have been disseminated worldwide and are now endemic in many hospitals globally (4, 5). Metallo-β-lactamases (MBLs) have also spread internationally. New Delhi MBL (NDM) is the most common MBL (6). Isolates producing class D OXA-48 carbapenemases are also increasingly common in Europe, although these isolates are less common in the United States (6, 7). Some CRE isolates do not produce a known carbapenemase and are referred to as non-carbapenemase-producing CRE (nonCP CRE) (8, 9). These isolates usually produce multiple extended-spectrum serine β-lactamases (ESBLs), may have increased expression of chromosomal cephalosporinases (AmpC), and/or possess outer membrane porin protein dysfunction (10).

Enterobacter species are among the species that constitutively produce AmpC enzymes. Among Enterobacter species, the Enterobacter cloacae complex, which contains E. cloacae, Enterobacter asburiae, Enterobacter hormaechei, Enterobacter kobei, Enterobacter ludwigii, Enterobacter mori, and Enterobacter nimipressuralis, includes the most common species that cause human infections (11). Therapeutic options for the treatment of E. cloacae complex include cefepime or carbapenems (12). In addition to the acquisition of KPC, resistance to cefepime and meropenem in E. cloacae can occur due to combinations of resistance mechanisms, including the overexpression of chromosomal AmpC, alterations in the outer membrane porin proteins, and/or upregulation of efflux systems, which limit the available treatment choices for these isolates.

In response to increasing numbers of CRE strains, β-lactam-β-lactamase inhibitor combinations with activity against serine carbapenemases, such as meropenem-vaborbactam, ceftazidime-avibactam, and imipenem-cilastatin-relebactam, were developed and approved for use in the United States and Europe (13–15). Vaborbactam is a cyclic boronic acid β-lactamase inhibitor that was developed to inhibit Ambler class A serine carbapenemases, including KPCs, class C β-lactamases, and class D noncarbapenemase oxacillinases (16). When combined with meropenem, vaborbactam restores the activity of this carbapenem against KPC-producing isolates. Vaborbactam, like other currently approved β-lactamase inhibitors, has no activity against class B MBLs (16–18). Meropenem-vaborbactam has been approved by the U.S. Food and Drug Administration (FDA) for the treatment of complicated urinary tract infections (cUTIs), including acute pyelonephritis, caused by Escherichia coli, K. pneumoniae, and E. cloacae complex in adults (15). The European Medicines Agency (EMA) has approved this agent also for the treatment of both hospital-acquired pneumonia (HAP) and ventilator-associated pneumonia (VAP), complicated intra-abdominal infections, and bacteremia associated with one of these urinary or nonurinary sources (14). The meropenem-vaborbactam breakpoints set by the Clinical and Laboratory Standards Institute (CLSI) and the U.S. FDA (susceptible, ≤4 mg/L; intermediate, 8 mg/L; resistant, ≥16 mg/L) reflect the higher meropenem dose of 2 g every 8 h used in combination with vaborbactam, while the lower breakpoints for meropenem alone (susceptible, ≤1 mg/L; intermediate, 2 mg/L; resistant, ≥4 mg/L) are based on a dose of 1 g every 8 h (19, 20).

In this study, we examined the susceptibility of 1,697 U.S. *Enterobacterales* isolates that were classified as multidrug resistant (MDR) (nonsusceptible to at least one agent in three or more drug classes) according to the method of Magiorakos et al. applying CLSI 2022 breakpoints (19, 21).

## RESULTS

### Organisms.

**FIG 1:**
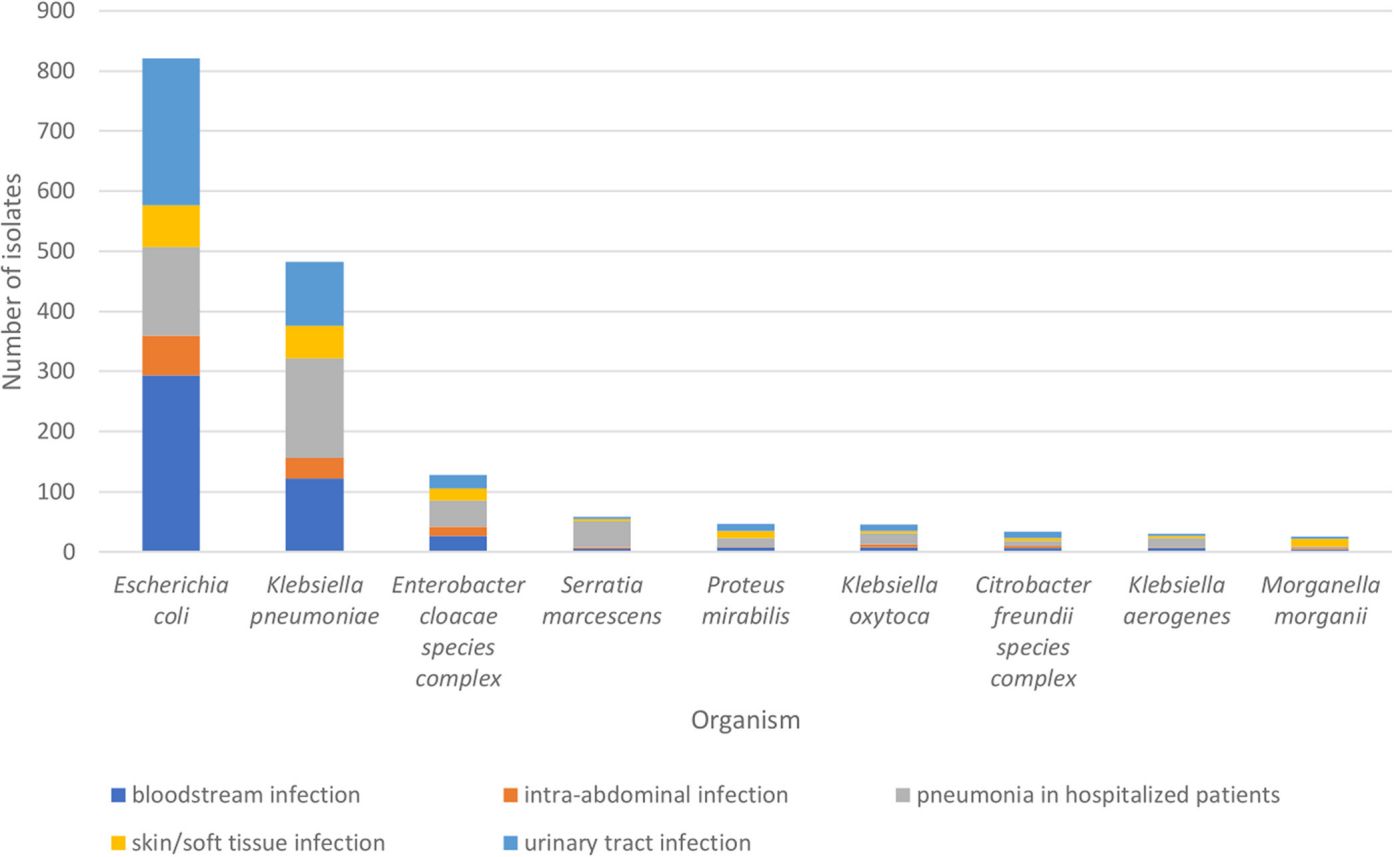
Species with >10 isolates, according to infection type.

A total of 23,359 *Enterobacterales* isolates were collected in 2016 to 2020 from U.S. medical centers by the SENTRY Antimicrobial Surveillance Program (22). Of those isolates, 1,697 (7.3%) were MDR and included in this study. The isolates were collected from hospitalized patients in 34 medical centers in all nine U.S. Census divisions. The Middle Atlantic division had the greatest number of MDR isolates, with 566 (33.3%) (see Fig. S1 in the supplemental material). The most common infection types with MDR isolates were bloodstream infections (*n *= 480) and pneumonia in hospitalized patients (*n *= 473), followed by urinary tract infections (*n *= 414) (Fig. 1). The most common organism isolated was Escherichia coli (*n *= 821), followed by K. pneumoniae (*n *= 483) and Enterobacter cloacae complex (*n *= 128).

### Susceptibility.

The rate of susceptibility of all MDR *Enterobacterales* strains to meropenem-vaborbactam was 99.1%, and 86.2% of the strains were susceptible to meropenem (Table 1). When the susceptibility of the three most common species was analyzed, rates of susceptibility to meropenem-vaborbactam were 96.9%, 99.8%, and 99.0% for E. cloacae complex, E. coli, and K. pneumoniae, respectively (Table 1). Rates of susceptibility to meropenem were 71.9%, 97.8%, and 73.5% for E. cloacae complex, E. coli, and K. pneumoniae, respectively. Similar rates of susceptibility to ceftazidime-avibactam were observed, i.e., 96.8%, 99.5%, and 99.2% for E. cloacae complex, E. coli, and K. pneumoniae, respectively. Resistance to other comparators was as expected for MDR isolates, with 7.5% of the isolates being susceptible to levofloxacin, 31.0% being susceptible to piperacillin-tazobactam, and 34.4% being susceptible to gentamicin. The most active non-β-lactam comparators overall were tigecycline (93.8% susceptible) and amikacin (92.6% susceptible) (Table 1). Rates of susceptibility to tigecycline were 85.2%, 100.0%, and 91.7% for E. cloacae complex, E. coli, and K. pneumoniae, respectively.

**TABLE 1:** Activity of meropenem-vaborbactam and comparator antimicrobials against MDR *Enterobacterales* strains, including Enterobacter cloacae complex, Escherichia coli, and Klebsiella pneumoniae isolates

Species and antimicrobial agent	MIC (mg/L)			Result with CLSI criteria (%)^*a*^			Result with U.S. FDA criteria (%)^*a*^		
	MIC_50_	MIC_90_	MIC range	S	I	R	S	I	R
MDR *Enterobacterales* (*n *= 1,697)									
Meropenem-vaborbactam	0.03	0.12	≤0.015 to >32	99.1	0.4	0.5	99.1^*b*^	0.4	0.5
Meropenem	0.03	4	≤0.015 to >32	86.2	2.4	11.5	86.2^*b*^	2.4	11.5
Imipenem	≤0.12	4	≤0.12 to >8	82	5.2	12.8	82.0^*b*^	5.2	12.8
Minocycline	4	32	0.25 to >32	64.9	10.5	24.6	64.9^*b*^	10.5	24.6
Tigecycline	0.5	2	≤0.06 to >8				93.8	5.2	1
Amikacin	4	16	≤0.25 to >32	92.6	5	2.4	92.6^*b*^	5	2.4
Cefepime	>16	>16	≤0.12 to >16	16.8^*c*^	11.7	71.5	16.8	11.7	71.5
Ceftazidime	32	>32	0.06 to >32	14.8	6.5	78.6	14.8^*b*^	6.5	78.6
Ceftazidime-avibactam (*n *= 1,310)^*d*^	0.25	1	≤0.015 to >32	99.1		0.9	99.1^*b*^		0.9
Colistin	0.25	4	≤0.06 to >8		89.9	10.1			
Gentamicin	>8	>8	≤0.12 to >8	34.4	5.8	59.8	34.4^*b*^	5.8	59.8
Levofloxacin	>4	>4	≤0.03 to >4	7.5	8.8	83.7	7.5^*b*^	8.8	83.7
Piperacillin-tazobactam	16	>64	≤0.5 to >64	31.0	21.0	48.0	52.0	19.6	28.4
E. cloacae complex (*n *= 128)									
Meropenem-vaborbactam	0.03	0.12	≤0.015 to >32	96.9	1.6	1.6	96.9^*b*^	1.6	1.6
Meropenem	0.12	8	≤0.015 to >32	71.9	3.9	24.2	71.9^*b*^	3.9	24.2
Imipenem	0.5	8	≤0.12 to >8	68	6.2	25.8	68.0^*b*^	6.2	25.8
Minocycline	8	32	0.5 to >32	43.8	17.2	39.1	43.8^*b*^	17.2	39.1
Tigecycline	1	4	0.12 to 4				85.2	14.8	0
Amikacin	2	8	0.5 to >32	96.1	1.6	2.3	96.1^*b*^	1.6	2.3
Cefepime	8	>16	0.12 to >16	30.5^*c*^	21.1	48.4	30.5	21.1	48.4
Ceftazidime	>32	>32	1 to >32	1.6	3.9	94.5	1.6^*b*^	3.9	94.5
Ceftazidime-avibactam (*n *= 93)	0.5	2	≤0.015 to >32	96.8		3.2	96.8^*b*^		3.2
Colistin	0.12	>8	≤0.06 to >8		89.0	11.0			
Gentamicin	4	>8	≤0.12 to >8	52.3	9.4	38.3	52.3^*b*^	9.4	38.3
Levofloxacin	4	>4	≤0.03 to >4	11.8	14.2	74	11.8^*b*^	14.2	74
Piperacillin-tazobactam	>64	>64	2 to >64	7.9	14.2	78	22.0	22.8	55.1
E. coli (*n *= 821)									
Meropenem-vaborbactam	0.03	0.03	≤0.015 to 32	99.8	0.0	0.2	99.8^*b*^	0.0	0.2
Meropenem	0.03	0.06	≤0.015 to 32	97.8	0.7	1.5	97.8^*b*^	0.7	1.5
Imipenem	≤0.12	0.25	≤0.12 to >8	97.8	1.0	1.2	97.8^*b*^	1.0	1.2
Minocycline	2	16	0.25 to >32	79.8	6.6	13.6	79.8^*b*^	6.6	13.6
Tigecycline	0.25	0.5	≤0.06 to 2				100.0	0.0	0.0
Amikacin	4	16	0.5 to >32	95.1	3.3	1.6	95.1^*b*^	3.3	1.6
Cefepime	>16	>16	≤0.12 to >16	11.8^*c*^	7.1	81.1	11.8	7.1	81.1
Ceftazidime	32	>32	0.12 to >32	16.7	7.4	75.9	16.7^*b*^	7.4	75.9
Ceftazidime-avibactam (*n *= 641)	0.25	0.5	≤0.015 to >32	99.5		0.5	99.5^*b*^		0.5
Colistin	0.12	0.25	≤0.06 to >8		99.8	0.2			
Gentamicin	>8	>8	0.25 to >8	29.5	0.9	69.7	29.5^*b*^	0.9	69.7
Levofloxacin	>4	>4	≤0.03 to >4	1.7	2.2	96.1	1.7^*b*^	2.2	96.1
Piperacillin-tazobactam	16	>64	0.5 to >64	44.8	23.8	31.5	68.5	17.4	14.0
K. pneumoniae (*n *= 483)									
Meropenem-vaborbactam	0.03	0.25	≤0.015 to >32	99.0	0.4	0.6	99.0^*b*^	0.4	0.6
Meropenem	0.03	16	≤0.015 to >32	73.5	4.1	22.4	73.5^*b*^	4.1	22.4
Imipenem	≤0.12	>8	≤0.12 to >8	75.6	1	23.4	75.6^*b*^	1	23.4
Minocycline	4	>32	0.5 to >32	58.8	12.4	28.8	58.8^*b*^	12.4	28.8
Tigecycline	0.5	2	≤0.06 to 8				91.7	6.8	1.4
Amikacin	4	32	≤0.25 to >32	86.1	9.1	4.8	86.1^*b*^	9.1	4.8
Cefepime	>16	>16	≤0.12 to >16	9.3^*c*^	7.3	83.4	9.3	7.3	83.4
Ceftazidime	>32	>32	0.12 to >32	5.0	4.3	90.7	5.0^*b*^	4.3	90.7
Ceftazidime-avibactam (*n *= 381)	0.25	1	≤0.015 to >32	99.2		0.8	99.2^*b*^		0.8
Colistin	0.25	0.25	≤0.06 to >8		94.3	5.7			
Gentamicin	>8	>8	≤0.12 to >8	33.1	9.9	56.9	33.1^*b*^	9.9	56.9
Levofloxacin	>4	>4	0.03 to >4	11.2	15.3	73.5	11.2^*b*^	15.3	73.5
Piperacillin-tazobactam	32	>64	0.5 to >64	14.7	23.4	61.9	38.1	21.5	40.4

a Criteria were published by the CLSI (19) and the U.S. FDA (20). S, sensitive; I, intermediate; R, resistant.

b The CLSI M100 standard is recognized (19).

c Intermediate is interpreted as susceptible-dose dependent.

d Ceftazidime-avibactam was not tested in 2016.

### MDR CRE.

**FIG 2:**
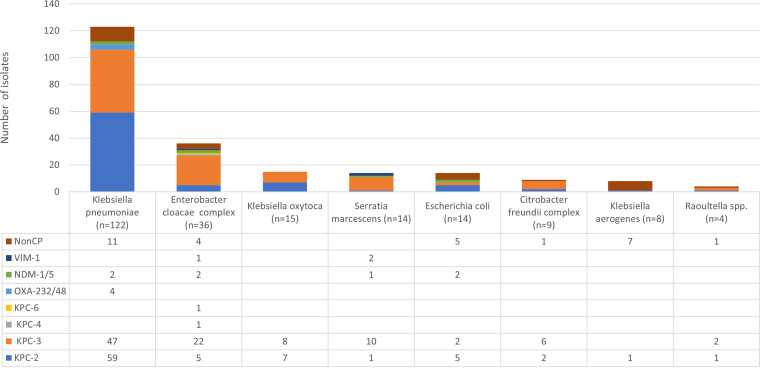
CRE strains according to species (number of each organism is shown), carbapenemases produced, and nonCP CRE. One K. pneumoniae isolate had both OXA-232 and NDM-1. No other isolates contained more than one carbapenemase.

There were 222 MDR CRE strains (13.1% of the MDR strains and 1.0% of the total *Enterobacterales* strains) that were defined as having MIC values of >2 mg/L for imipenem and/or meropenem. CRE strains were submitted from centers in all nine U.S. Census divisions. The Middle Atlantic division had the greatest number of MDR CRE strains (*n *= 119 [53.6%]), while the West North Central division had no CRE strains (see Fig. S1). K. pneumoniae (*n *= 122) was the most common CRE species, with 25.3% of MDR K. pneumoniae strains also being CRE (Fig. 2). The second most common MDR CRE species was E. cloacae complex (*n *= 36), with 28.1% of MDR E. cloacae complex strains testing as CRE. Only 1.7% (*n *= 14) of MDR E. coli strains were CRE.

KPC was the most common carbapenemase produced (81.1%), with 81 strains producing KPC-2 (including 2 producing KPC-2-like carbapenemase) and 97 producing KPC-3 (Fig. 2). Overall, 58.9% of KPC producers were K. pneumoniae strains, while 16.1% were E. cloacae complex strains. MBLs and OXA-48-like carbapenemases were uncommon, with 7 isolates (3.2%) producing NDM, 3 isolates (1.4%) producing VIM, and 3 isolates (1.4%) producing OXA-48-like carbapenemase alone; 1 K. pneumoniae isolate coproduced both NDM-1 and OXA-232 (Fig. 2).

**FIG 3:**
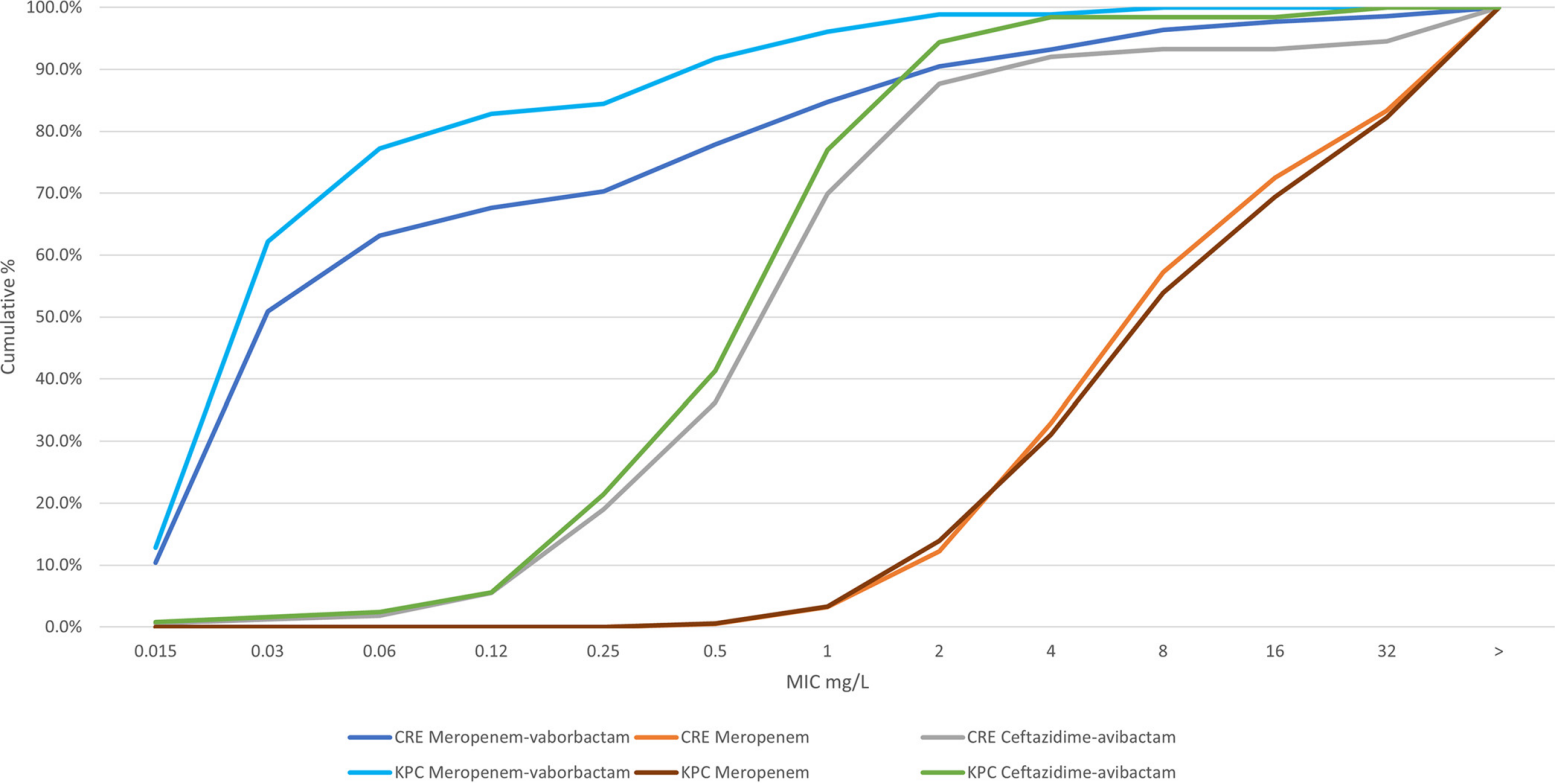
Cumulative percentages of MIC values for all CRE- and KPC-containing isolates for meropenem-vaborbactam, meropenem, and ceftazidime-avibactam.

The cumulative percentages of MIC distributions for meropenem, meropenem-vaborbactam, and ceftazidime-avibactam against all CRE and KPC-producing isolates are shown in Fig. 3. Susceptibilities of CRE, KPC, and nonCP CRE subsets to all comparators are shown in Table 2. The rate of susceptibility of all CRE strains to meropenem-vaborbactam was 93.2%, while the rate of susceptibility of the KPC-producing isolates was 98.9%. Rates of susceptibility to ceftazidime-avibactam were similar, i.e., 93.3% for all CRE strains and 98.4% for KPC-producing isolates (Table 2). Although rates of susceptibility were similar, meropenem-vaborbactam was more potent than ceftazidime-avibactam against CRE strains, with MIC_50_ and MIC_90_ values of 0.03 and 2 mg/L, respectively, for meropenem-vaborbactam, compared to MIC_50_ and MIC_90_ values of 1 and 4 mg/L, respectively, for ceftazidime-avibactam (Fig. 3). Meropenem-vaborbactam was also more potent than ceftazidime-avibactam against KPC-producing isolates, with MIC_50_ and MIC_90_ values of 0.03 and 0.5 mg/L, respectively, for meropenem-vaborbactam, compared to MIC_50_ and MIC_90_ values of 1.0 and 2.0 mg/L, respectively, for ceftazidime-avibactam.

**TABLE 2:** Susceptibilities of MDR CRE strains to meropenem-vaborbactam and comparators and subsets for KPC-producing and non-carbapenemase-producing isolates

Strain type and antimicrobial agent	MIC (mg/L)			Result with CLSI criteria (%)^*a*^			Result with FDA criteria (%)^*a*^		
	MIC_50_	MIC_90_	MIC range	S	I	R	S	I	R
MDR CRE (*n *= 222)									
Meropenem-vaborbactam	0.03	2	≤0.015 to >32	93.2	3.2	3.6	93.2^*b*^	3.2	3.6
Meropenem	8	>32	0.5 to >32	3.2	9.0	87.8	3.2^*b*^	9.0	87.8
Imipenem	8	>8	0.5 to >8	5.0	2.3	92.8	5.0^*b*^	2.3	92.8
Minocycline	4	>32	0.5 to >32	63.5	12.6	23.9	63.5^*b*^	12.6	23.9
Tigecycline	0.5	2	≤0.06 to 8				95.5	2.7	1.8
Amikacin	4	32	0.5 to >32	78.8	14.4	6.8	78.8^*b*^	14.4	6.8
Cefepime	>16	>16	0.25 to >16	7.2	13.1^*c*^	79.7	7.2	13.1	79.7
Ceftazidime	>32	>32	1 to >32	2.3	4.1	93.7	2.3^*b*^	4.1	93.7
Ceftazidime-avibactam (*n *= 163)^*d*^	1	4	≤0.015 to >32	93.3		6.7	93.3^*b*^		6.7
Colistin	0.12	>8	≤0.06 to >8		87.7	12.3			
Gentamicin	8	>8	≤0.12 to >8	49.5	14.9	35.6	49.5^*b*^	14.9	35.6
Levofloxacin	>4	>4	≤0.03 to >4	18.0	5.4	76.6	18.0^*b*^	5.4	76.6
Piperacillin-tazobactam	>64	>64	32 to >64	0.0	0.0	100.0	0.0	5.4	94.6
KPC-producing (*n *= 180)									
Meropenem-vaborbactam	0.03	0.5	≤0.015 to 8	98.9	1.1	0.0	98.9^*b*^	1.1	0.0
Meropenem	8	>32	0.5 to >32	3.3	10.6	86.1	3.3^*b*^	10.6	86.1
Imipenem	>8	>8	1 to >8	0.6	0.6	98.9	0.6^*b*^	0.6	98.9
Minocycline	4	>32	0.5 to >32	64.4	11.1	24.4	64.4^*b*^	11.1	24.4
Tigecycline	0.5	2	≤0.06 to 8				96.1	1.7	2.2
Amikacin	4	32	0.5 to >32	76.1	17.8	6.1	76.1^*b*^	17.8	6.1
Cefepime	>16	>16	0.25 to >16	7.2	14.4^*c*^	78.3	7.2	14.4	78.3
Ceftazidime	>32	>32	1 to >32	2.8	5.0	92.2	2.8^*b*^	5.0	92.2
Ceftazidime-avibactam (*n *= 126)	1	2	≤0.015 to 32	98.4		1.6	98.4^*b*^		1.6
Colistin	0.25	>8	≤0.06 to >8		86.5	13.5			
Gentamicin	8	>8	≤0.12 to >8	46.7	18.3	35	46.7^*b*^	18.3	35
Levofloxacin	>4	>4	≤0.03 to >4	15	5.6	79.4	15.0^*b*^	5.6	79.4
Piperacillin-tazobactam	>64	>64	32 to >64	0.0	0.0	100.0	0.0	3.9	96.1
Non-CP CRE (*n *= 29)									
Meropenem-vaborbactam	2	8	0.03 to 16	89.7	6.9	3.4	89.7^*b*^	6.9	3.4
Meropenem	8	16	2 to >32	0.0	3.4	96.6	0.0^*b*^	3.4	96.6
Imipenem	4	>8	0.5 to >8	31	13.8	55.2	31.0^*b*^	13.8	55.2
Minocycline	4	>32	0.5 to >32	58.6	17.2	24.1	58.6^*b*^	17.2	24.1
Tigecycline	0.5	4	0.12 to 4				89.7	10.3	0.0
Amikacin	2	8	0.5 to 16	100.0	0.0	0.0	100.0^*b*^	0.0	0.0
Cefepime	>16	>16	2 to >16	10.3^*c*^	10.3	79.3	10.3	10.3	79.3
Ceftazidime	>32	>32	16 to >32	0.0	0.0	100.0	0.0^*b*^	0.0	100
Ceftazidime-avibactam (*n *= 29)	1	4	0.12 to 8	100.0		0.0	100.0^*b*^		0.0
Colistin	0.12	0.25	≤0.06 to 0.5		100.0	0.0			
Gentamicin	1	>16	≤0.12 to >16	65.5	0.0	34.5	65.5^*b*^	0.0	34.5
Levofloxacin	4	>4	≤0.03 to >4	37.9	6.9	55.2	37.9^*b*^	6.9	55.2
Piperacillin-tazobactam	>64	>64	32 to >64	0.0	0.0	100.0	0.0	17.2	82.8

a Criteria were published by the CLSI (19) and the U.S. FDA (20). S, sensitive; I, intermediate; R, resistant.

b The CLSI M100 standard is recognized (19).

c Intermediate is interpreted as susceptible-dose dependent.

d Ceftazidime-avibactam was not tested in 2016.

Three isolates produced OXA-48 or OXA-232 alone; 1 was susceptible to meropenem-vaborbactam, 1 was intermediate, and 1 was resistant. All 3 isolates were susceptible to ceftazidime-avibactam. Of the 3 isolates producing VIM-1, 2 were meropenem-vaborbactam susceptible and 1 was intermediate; 1 was susceptible to ceftazidime-avibactam and 2 were resistant. Seven isolates produced NDM-like enzymes, including 1 isolate with both OXA-232 and NDM-1; all 7 were nonsusceptible to meropenem-vaborbactam and ceftazidime-avibactam.

There were 29 CRE isolates (13.1%) that had no described carbapenemase genes detected by whole-genome sequencing (WGS), i.e., nonCP CRE isolates (Fig. 2). All nonCP CRE isolates, except Klebsiella aerogenes isolates, produced multiple β-lactamases, including CTX-M, TEM, SHV, OXA-1, and/or plasmidic AmpC cephalosporinases (ACT-17 and CMY-2) (see Table S1). All except 1 isolate (Raoultella ornithinolytica) also had premature stop codons, insertions, and/or deletions in one or more of the outer membrane protein genes (*ompC*, *ompF*, *ompK35*, or *ompK36*), causing disruption or alteration of porin function. Seven K. aerogenes isolates had no acquired β-lactamases and only chromosomal AmpC and Omp dysfunction. The combination of multiple β-lactamases with outer membrane porin disruptions has been associated with carbapenem resistance (10). The susceptibilities of the nonCP CRE isolates are shown in Table 2 and Table S1. The rate of susceptibility to meropenem was 0%, that for imipenem was 31%, that for meropenem-vaborbactam was 89.7% (MIC_50_, 2 mg/L; MIC_90_, 8 mg/L), and that for ceftazidime-avibactam was 100% (MIC_50_, 1 mg/L; MIC_90_, 4 mg/L). Of the 3 meropenem-vaborbactam-nonsusceptible nonCP CRE isolates, 1 K. aerogenes isolate was resistant to meropenem-vaborbactam (MIC, 16 mg/L) and had a chromosomal AmpC and a disrupted OmpC. Two isolates, 1 K. aerogenes and 1 K. pneumoniae were intermediate to meropenem-vaborbactam (MIC, 8 mg/L). The K. aerogenes isolate produced a chromosomal AmpC, and the K. pneumoniae isolate produced CTX-M-15, OXA-1, OXA-30, SHV-168, and TEM-1 plus it had a disrupted OmpK36. The expression levels of AmpC were not determined. The cause of the elevated meropenem-vaborbactam MIC values for these 3 isolates requires future investigation. Ceftazidime-avibactam MIC values were 2 mg/L for the 2 K. aerogenes isolates and 8 mg/L for the K. pneumoniae isolate.

The most active non-β-lactam comparators against MDR CRE strains were tigecycline (95.5% susceptible) and amikacin (78.8% susceptible) (Table 2).

## DISCUSSION

This study provides information on the susceptibility to meropenem-vaborbactam of 1,697 MDR *Enterobacterales* isolates, including 222 MDR CRE strains, that were collected at medical centers in the United States in 2016 to 2020. The overall rate of susceptibility of MDR isolates to meropenem-vaborbactam and ceftazidime-avibactam was 99.1%, while the rate of susceptibility to meropenem was 86.2%. The overall rate of MDR in this study was 7.3%, lower than the MDR rate of 16.1% in a recent study of European medical centers by Sader et al. (23). The CRE rate was 1.0% of all *Enterobacterales* strains and 13.1% of this MDR population. This CRE rate was lower than the 3.6% rate reported for Europe overall and is more similar to the Western European CRE rate of 1.4%.

The primary carbapenemase in the present study was KPC (81.1%); the presence of MBLs (NDM and VIM) or OXA-48-like carbapenemases was uncommon (4.5% of isolates produced MBLs and 1.4% produced OXA-48-like carbapenemases). This is in contrast to findings for Europe, where MBL- and OXA-48-producing isolates are increasingly observed, particularly in Eastern European countries, where OXA-48 is becoming as common as KPC (6, 23–25).

The nonCP CRE frequency in our study was 13.1% of the CRE cohort, similar to the results of a recent study by Castanheira et al. on isolates from 71 U.S. hospitals, which identified a nonCP CRE frequency of 14.8% (10), but in contrast to the results of Tamma et al. on isolates from three U.S. hospitals, which identified a nonCP CRE frequency of 54% (26). It is possible that the high nonCP CRE rate reported by Tamma et al. (26) represents a local clonal spread. The nonCP CRE rate for European CRE strains was 17.7% in the study by Sader et al. (23), with Poland having a greater number than other European countries in additional recent studies (24, 27).

The three main MDR species were E. cloacae complex, E. coli, and K. pneumoniae. E. coli was the most common MDR species, but only 1.7% of the strains were CRE. Klebsiella pneumoniae was the second most common MDR species and the most common CRE species, with the majority of strains producing KPC. E. cloacae complex was the third most common MDR species and the second most common CRE species; the main carbapenemase produced by these isolates was also KPC. The meropenem-vaborbactam susceptibility rates were >96% for all three organisms. The two antimicrobials often considered for treatment of infections caused by E. cloacae complex, cefepime and meropenem, had susceptibility rates of 30.5% and 71.9%, respectively. These results suggest that, in a region with a large MDR population, cefepime would not be a useful therapeutic choice; meropenem may be useful, depending on the local rate of carbapenem-resistant organisms.

Our study has several limitations. First, isolates were considered the most probable cause of an infection by the submitting site, but no chart review was performed. Second, hospitals were asked to send only 1 isolate per patient per infection episode; however, the possibility that isolates were received from the same patient during separate hospitalizations cannot be ruled out. Third, WGS identified that the nonCP CRE isolates did not have recognized carbapenemase genes, but there is a possibility that an unrecognized carbapenemase was present.

In summary, the primary carbapenemase in the United States continues to be KPC, while MBLs and OXA-48-like carbapenemases remain uncommon. Overall, these U.S. MDR organisms demonstrated a meropenem-vaborbactam susceptibility rate of 99.1%, and thus meropenem-vaborbactam remains a useful treatment for Gram-negative infections caused by MDR organisms.

## MATERIALS AND METHODS

Participating medical centers, located in all nine U.S. Census divisions, were asked to submit 1 isolate per patient per infection episode, according to a common protocol of the SENTRY Antimicrobial Surveillance Program (22). Each isolate was considered the probable cause of the infection by the submitting site. No medical chart reviews were performed.

Isolates were identified at the submitting site and confirmed at JMI Laboratories by matrix-assisted laser desorption ionization–time of flight mass spectrometry (MALDI-TOF MS) using a MALDI Biotyper (Bruker Daltonics, Billerica, MA, USA). Susceptibility testing was performed using the CLSI broth microdilution method with frozen form panels produced at JMI Laboratories (28). CLSI and U.S. FDA interpretive criteria were used (19, 20). CLSI quality control organisms were tested as appropriate for the tested agents, and all results were within the specified ranges. Piperacillin-tazobactam and ceftazidime-avibactam were tested with a fixed concentration of inhibitor of 4 mg/L, while meropenem-vaborbactam was tested with a fixed concentration of inhibitor of 8 mg/L.

Isolates were classified as MDR (resistant to at least one antimicrobial in three or more drug classes) according to the method of Magiorakos et al. using CLSI 2022 breakpoints (20). CRE strains were classified as having MIC values of >2 mg/L for imipenem and/or meropenem (19). Imipenem MIC values were not used to categorize Proteus, *Providencia*, or *Morganella* spp.

WGS was performed for each CRE isolate as described previously (29, 30). Known carbapenemase genes, including *bla*_KPC_, *bla*_NDM_, *bla*_IMP_, *bla*_VIM_, and *bla*_OXA-48-like_, were not detected in 29 CRE strains. The nonCP CRE sequences were further analyzed for the presence of other β-lactamases and disruption/alteration in outer membrane porin proteins (OmpC, OmpF, OmpK35, OmpK36, and OmpK37), as described previously (29).
