# Supplementary material for: Meropenem-Vaborbactam Activity against U.S. Multidrug-Resistant Enterobacterales Strains, Including Carbapenem-Resistant Isolates
Source: Microbiol Spectr. 2023 Jan 9;11(1):e04507-22. doi: 10.1128/spectrum.04507-22 (PMC9927278; doi:10.1128/spectrum.04507-22)
Supplement: Supplemental file 1 — Supplemental material. Download spectrum.04507-22-s0001.pdf, PDF file, 0.1 MB [file spectrum.04507-22-s0001.pdf]

Supplemental Figure 1. MDR and CRE by US Census Division.

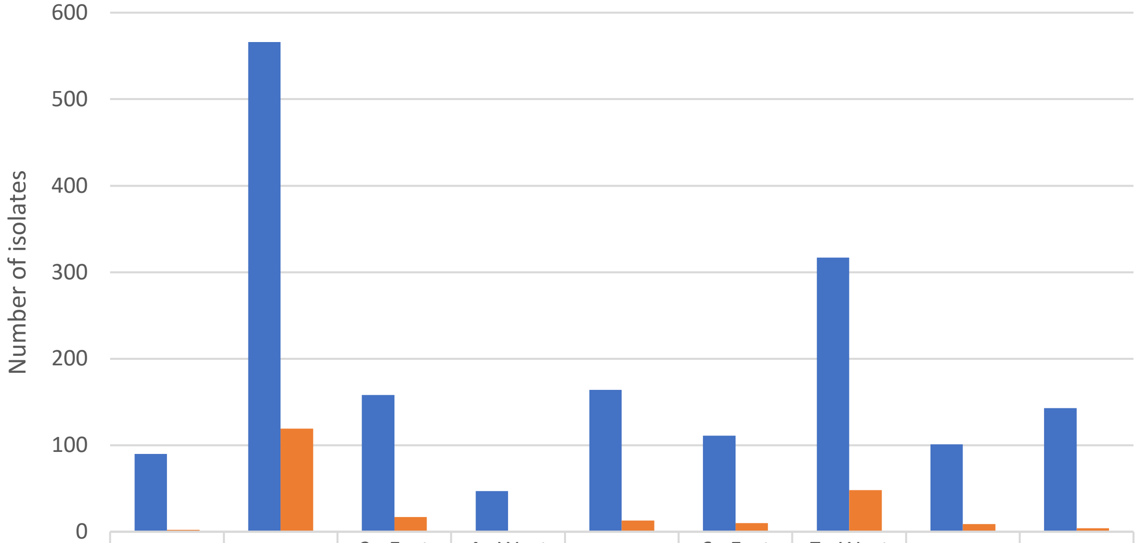

	1: New England	2: Middle Atlantic	3: East North Central	4: West North Central	5: South Atlantic	6: East South Central	7: West South Central	8: Mountain	9: Pacific
MDR	90	566	158	47	164	111	317	101	143
CRE	2	119	17	0	13	10	48	9	4

Supplemental Table 1. List of nonCP CRE organisms with resistance mechanisms, and carbapenem, meropenem-vaborbactam, and ceftazidime-avibactam MIC values

Organism	Study Year	State	Meropenem-vaborbactam	Meropenem	Imipenem	Ceftazidime-avibactam	Beta-lactamase	OmpC/OmpK36	OmpF/OmpK35
Citrobacter freundii complex	2018	TX	4	8	8	4	CMY-48-like, TEM-1	disrupted	alterations
Enterobacter cloacae complex	2016	CO	4	8	8	1	ACT-17	disrupted	alterations
Enterobacter cloacae complex	2016	NY	2	8	4	1	ACT-25	disrupted	disrupted
Enterobacter cloacae complex	2017	NY	0.5	8	2	1	ACT-17	disrupted	alterations
Enterobacter cloacae complex	2018	KY	0.5	4	4	1	ACT-2	disrupted	disrupted
Escherichia coli	2016	MI	0.5	4	1	0.12	CTX-M-15, TEM-1	disrupted	alterations
Escherichia coli	2017	TX	2	8	2	1	CTX-M-14	alterations	alterations
Escherichia coli	2018	LA	0.25	2	4	0.25	CMY-2, EC-6, TEM-1	disrupted	alterations
Escherichia coli	2018	FL	0.25	4	1	0.12	CTX-M-15, EC-6	disrupted	alterations
Escherichia coli	2019	TX	0.25	4	1	0.25	CTX-M-15, EC-6, TEM-1	alterations	wild-type
Klebsiella aerogenes	2016	NJ	1	8	8	1	ampC	disrupted	disrupted
Klebsiella aerogenes	2016	TX	1	8	4	0.5	ampC	disrupted	alterations
Klebsiella aerogenes	2018	CO	1	4	8	0.5	ampC	disrupted	disrupted
Klebsiella aerogenes	2019	TX	1	4	8	0.5	ampC	disrupted	alterations
Klebsiella aerogenes	2019	CO	2	8	>8	0.5	ampC	disrupted	alterations
Klebsiella aerogenes	2020	TX	16	16	>8	2	ampC	disrupted	disrupted
Klebsiella aerogenes	2020	NY	8	16	>8	2	ampC	alterations	alterations
Klebsiella pneumoniae	2017	NY	1	4	1	1	CTX-M-15	disrupted	alterations
Klebsiella pneumoniae	2017	TX	4	4	2	2	CTX-M-15	disrupted	wild-type

Organism	Study Year	State	Meropenem-vaborbactam	Meropenem	Imipenem	Ceftazidime-avibactam	Beta-lactamase	OmpC/OmpK36	OmpF/OmpK35
Klebsiella pneumoniae	2018	NY	2	4	1	1	CTX-M-15, OXA-1_OXA-30, SHV-11, TEM-1	disrupted	alterations
Klebsiella pneumoniae	2018	FL	2	8	4	0.12	CTX-M-15, OXA-1_OXA-30, SHV-11	disrupted	alterations
Klebsiella pneumoniae	2019	MA	2	8	1	4	CTX-M-15, SHV-11	alterations	alterations
Klebsiella pneumoniae	2019	LA	0.03	16	>8	2	CTX-M-15, OXA-1_OXA-30, SHV-11	alterations	disrupted
Klebsiella pneumoniae	2019	TX	2	4	1	1	CTX-M-15, OXA-1_OXA-30, SHV-187, TEM-1	disrupted	disrupted
Klebsiella pneumoniae	2019	TX	2	4	0.5	1	CTX-M-71, SHV-11, TEM-1	disrupted	wild-type
Klebsiella pneumoniae	2019	VA	0.5	4	0.5	1	CTX-M-15, OXA-1_OXA-30, SHV-11, TEM-1	disrupted	disrupted
Klebsiella pneumoniae	2019	NY	4	8	2	2	CTX-M-15, OXA-1_OXA-30, SHV-28, TEM-1	disrupted	alterations
Klebsiella pneumoniae	2020	NY	8	16	4	8	CTX-M-15, OXA-1_OXA-30, SHV-168, TEM-1	disrupted	wild-type
Raoutella ornithinolytica	2020	NY	1	>32	>8	8	CARB-2_PSE-1, FOX-5, ORN-1	wild-type	wild-type
